# Observation of unexpected uniaxial magnetic anisotropy in La_2/3_Sr_1/3_MnO_3_ films by a BaTiO_3_ overlayer in an artificial multiferroic bilayer

**DOI:** 10.3762/bjnano.11.51

**Published:** 2020-04-16

**Authors:** John E Ordóñez, Lorena Marín, Luis A Rodríguez, Pedro A Algarabel, José A Pardo, Roger Guzmán, Luis Morellón, César Magén, Etienne Snoeck, María E Gómez, Manuel R Ibarra

**Affiliations:** 1 Department of Physics, Universidad del Valle, A.A. 25360, Cali, Colombia; 2 Departamento de Física de la Materia Condensada, Universidad de Zaragoza, 50009 Zaragoza, Spain; 3 Instituto de Nanociencia de Aragón (INA), Universidad de Zaragoza, 50018 Zaragoza, Spain; 4 Centro de Excelencia de Nuevos Materiales (CENM), Universidad del Valle, A.A. 25360, Cali, Colombia; 5 Laboratorio de Microscopías Avanzadas (LMA)-Instituto de Nanociencia de Aragón (INA), Universidad de Zaragoza, 50018 Zaragoza, Spain; 6 CEMES-CNRS, 29 rue Jeanne Marvig, B.P. 94347, F-31055 Toulouse Cedex, France; 7 Instituto de Ciencia de Materiales de Aragón (ICMA), CSIC-Universidad de Zaragoza, 50009 Zaragoza, Spain; 8 Departamento de Ciencia y Tecnología de Materiales y Fluidos, Universidad de Zaragoza, 50018 Zaragoza, Spain

**Keywords:** artificial multiferroic system, BaTiO_3_, interface-induced strain, La_2/3_Sr_1/3_MnO_3_, magnetic anisotropy

## Abstract

We studied in detail the in-plane magnetic properties of heterostructures based on a ferroelectric BaTiO_3_ overlayer deposited on a ferromagnetic La_2/3_Sr_1/3_MnO_3_ film grown epitaxially on pseudocubic (001)-oriented SrTiO_3_, (LaAlO_3_)_0.3_(Sr_2_TaAlO_6_)_0.7_ and LaAlO_3_ substrates. In this configuration, the combination of both functional perovskites constitutes an artificial multiferroic system with potential applications in spintronic devices based on the magnetoelectric effect. La_2/3_Sr_1/3_MnO_3_ single layers and BaTiO_3_/La_2/3_Sr_1/3_MnO_3_ bilayers using the pulsed-laser deposition technique. We analyzed the films structurally through X-ray reciprocal space maps and high-angle annular dark field microscopy, and magnetically via thermal demagnetization curves and in-plane magnetization versus applied magnetic field loops at room temperature. Our results indicate that the BaTiO_3_ layer induces an additional strain in the La_2/3_Sr_1/3_MnO_3_ layers close to their common interface. The presence of BaTiO_3_ on the surface of tensile-strained La_2/3_Sr_1/3_MnO_3_ films transforms the in-plane biaxial magnetic anisotropy present in the single layer into an in-plane uniaxial magnetic anisotropy. Our experimental evidence suggests that this change in the magnetic anisotropy only occurs in tensile-strained La_2/3_Sr_1/3_MnO_3_ film and is favored by an additional strain on the La_2/3_Sr_1/3_MnO_3_ layer promoted by the BaTiO_3_ film. These findings reveal an additional mechanism that alters the magnetic behavior of the ferromagnetic layer, and consequently, deserves further in-depth research to determine how it can modify the magnetoelectric coupling of this hybrid multiferroic system.

## Introduction

In recent years, enormous interest has been shown in the multiferroic properties of the multilayered system based on La_2/3_Sr_1/3_MnO_3_ (LSMO) and BaTiO_3_ (BTO) films [1–5]. Each perovskite material has a particular ferroic order at room temperature, i.e., ferromagnetic (FM) for LSMO and ferroelectric (FE) for BTO, and BTO/LSMO heterostructures have exhibited magnetoelectric coupling (MEC) [6–8]. They constitute a type of artificial hybrid multiferroic material that can be employed to build the next-generation sensors, multiple-state memory elements, magnetic read/write hard disks, actuators, etc. [9–10]. In multilayered films, both electrical and magnetic properties of these ferroic perovskites are strongly affected by crystal distortions originated by lattice-misfit strain at the film/substrate interface [11–14]. For LSMO and other manganites, the effect of the substrate-induced strain on its magnetic properties in single-layer configuration has been widely studied, particularly in regards to the influence of strain on the magnetic anisotropy [15–16]. Depending on the type and magnitude of the imposed biaxial strain (compressive or tensile) [17], the magnetic anisotropy of LSMO film can be altered in different ways: (i) to give rise to the appearance of a uniaxial in-plane magnetic anisotropy contribution, which is significantly stronger than the cubic one [15,18–21], (ii) to induce an out-of-plane magnetic anisotropy in compressive-strained films [22–26], or (iii) to suppress the FM ordering in a small region of the layer due to large crystal deformations, resulting in the formation of a dead layer with antiferromagnetic-insulating behavior [12,27–31]. Moreover, it was found that a uniaxial magnetic anisotropy is artificially induced in LSMO films grown on ferroelectric BiFeO_3_ substrate when the polarization of the FE domains is switched to highly aligned stripe domains, inducing a magnetic easy axis in the FM layer parallel to the polarization direction [32–33]. In all aforementioned cases, the magnetic anisotropy of LSMO is also affected either by a non-FE or FE substrate on which it is deposited. However, an open question remains about how the presence of a FE-BTO layer grown on top of an FM-LSMO film can alter its magnetic properties. This is a key point that needs to be evaluated to improve our understanding of the mechanisms driving MEC in BTO/LSMO heterostructures.

In this work, we show how the presence of a BTO layer on an LSMO film under different substrate-induced epitaxial strains can affect the magnetic anisotropy of the LSMO layer at room temperature. To strain the sample, we epitaxially have grown BTO/LSMO bilayers on SrTiO_3_ (STO), (LaAlO_3_)_0.3_(Sr_2_TaAlO_6_)_0.7_ (LSAT) and LaAlO_3_ (LAO) single-crystal substrates where we choose for all of them the pseudocubic (001) direction perpendicular the substrate surface. We have grown the samples by pulsed-laser deposition and systematically varied the layer thicknesses. We structurally analyzed samples by reciprocal space maps (RSMs) around the pseudocubic (103) reflection in an X-ray diffractometer and high-angle annular dark field in scanning transmission electron microscopy (HAADF-STEM). Local strain maps were reconstructed by the geometric phase analysis (GPA) method on HAADF-STEM images. We magnetically analyzed samples by performing room-temperature polar plots of the remnant field, where we applied magnetic field on the plane of the sample along different directions.

## Results and Discussion

**Figure 1:**
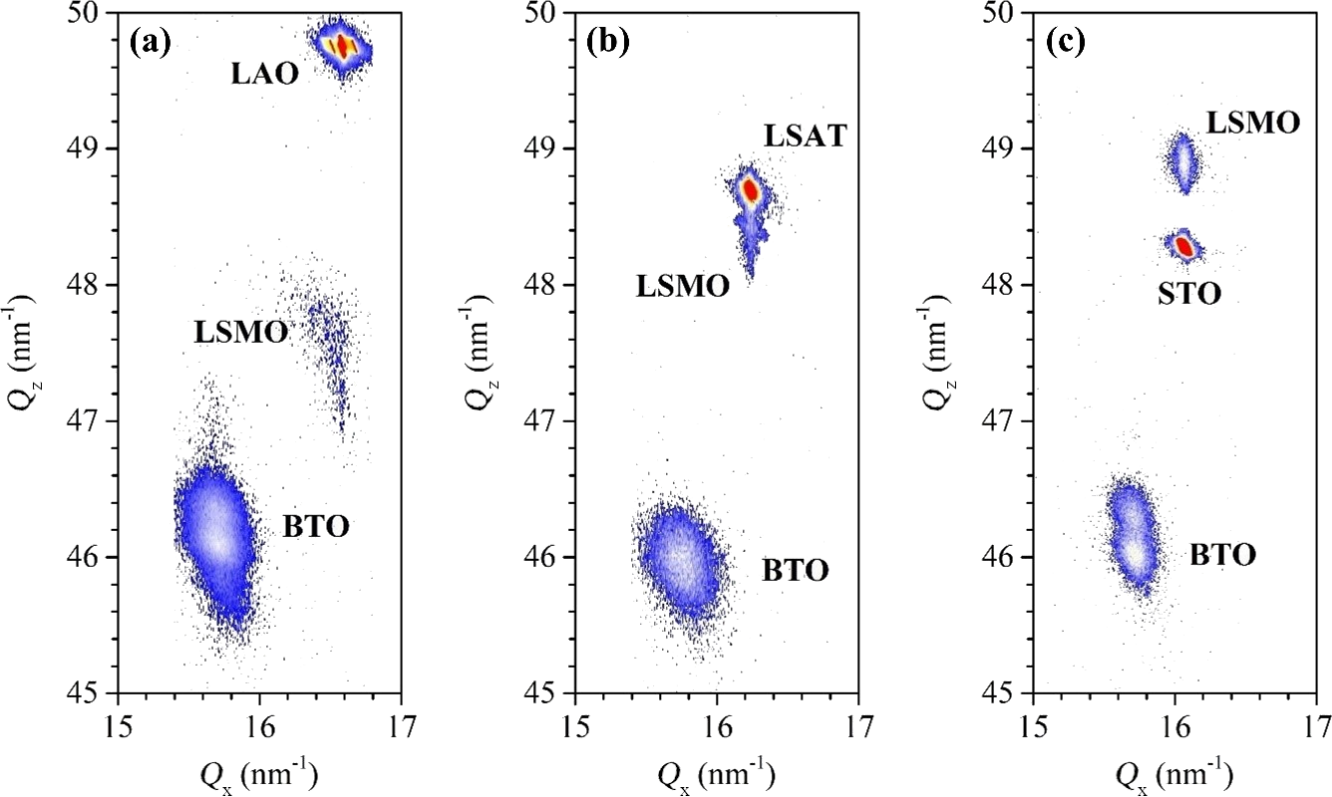
X-ray reciprocal space maps around the pseudo-cubic (103) Bragg reflection for the BTO/LSMO bilayers grown on (a) LAO, (b) LSAT, and (c) STO substrates.

Figure 1 displays RSMs taken around the pseudocubic (103) reflection for the BTO (140 nm)/LSMO (27 nm) bilayers grown on (001)-oriented LAO (a), LSAT (b), and STO (c) substrates. The maps exhibit three main irregular spots corresponding to the pseudocubic (103) reflection of each material present in the heterostructures: BTO, LSMO, and substrate. We plotted the out-of-plane component of the scattering vector, *Q*_z_ (growth direction), versus its in-plane component, *Q*_x_ (associated with the [100] direction). In all cases, we noted that for the LSMO reflections, the position of *Q*_x_ coincides (within the measurement margin of error) with those of the substrates, while the position of *Q*_z_ is quite different from that of each substrate, being almost superimposed for LSMO grown on LSAT (see Figure 1b). This behavior corroborates the expected fully strained epitaxial growth (cube-on-cube) of the LSMO film where its in-plane lattice parameter is adapted to that of the substrate, and its out-of-plane lattice parameter is deformed accordingly [34–35]. The LSMO reflection for the sample grown on LAO substrate, Figure 1a, exhibits a low-intensity broad spot, which is influenced by the twinned nature of the rhombohedral LAO substrate, evidenced by the splitting of the LAO main reflection into three spots.

For the BTO layers, its *Q*_x_ position is the same, within the experimental error, for all samples, and it does not coincide with the LSMO and substrate reflections, indicating that the in-plane lattice parameter of the BTO is not adapted to the substrate or to the LSMO film. Similarly, the *Q*_z_ position of the BTO reflection is also the same in all maps. Interestingly, we observed a clear splitting of the BTO reflection spot for the bilayers grown on STO and LAO substrates, indicating the possible two different out-of-plane lattice parameters.

From *Q*_x_ and *Q*_z_ scattering vector components of each reflection spot, we have calculated the in-plane and out-of-plane lattice parameters of the BTO and LSMO layers in the heterostructures. We summarized the results in Table 1. We also listed the lattice deformation (in percentage) due to the induced strain in the in-plane (*f*_a-system_) and out-of-plane (*f*_c-system_) lattice parameter of each film, defining *f*_a-system_ and *f*_c-system_ as:

[1] $$f_{\text{a-system}}=\left(\frac{a_{\text{film-system}}-a_{\text{bulk-system}}}{a_{\text{bulk-system}}}\right)\times 100$$

[2] $$f_{\text{c-system}}=\left(\frac{c_{\text{film-system}}-c_{\text{bulk-system}}}{c_{\text{bulk-system}}}\right)\times 100$$

where *a* and *c* correspond to the in-plane and out-of-plane lattice parameter of the system and the subscript film and bulk are associated to the lattice parameters measured in the film and that reported for the bulk of each material, respectively. According to Equation 1, positive (negative) values of *f*_a-system_ correspond to tensile (compressive) strain. In Table 1 we summarize the strain values. As expected from the LSMO and substrate mismatches, STO and LAO substrates induce a high compressive (*f*_a-LSMO_ = −2.3%) and moderate tensile (*f*_a-LSMO_ = +0.7%) strain, respectively. A very weak compressive strain (*f*_a-LSMO_ = −0.2%) is observed in the LSMO film grown on LSAT. According to Poisson’s effect, each tensile (compressive) in-plane strain led to shrinkage (elongation) of *c*_LSMO_, where the higher the *f*_a-LSMO_ magnitude the higher the *f*_c-LSMO_ magnitude. For the BTO layers, the in-plane lattice parameter is close to that of the bulk value, whereas the *c*_BTO_ reveals two values corresponding to the two spots observed in the RSMs (Figure 1).

**Table 1:** In- and out-of-plane lattice parameters, *c*/*a* ratio, and lattice deformation for BTO and LSMO layers in the heterostructures (bulk values at room temperature are: *a*_LSMO_ = *c*_LSMO_ = 3.876 Å [36], *a*_BTO_ = 3.999 Å, *c*_BTO_ = 4.033 Å [37]).

Substrate	Film	In-plane [Å]	Out-of-plane [Å]	*c*/*a* ratio	*f*_a_ [%]	*f*_c_ [%]

LAO	LSMO	3.80(1)	3.98(4)	1.05(1)	−2.3(3)	+2(1)
	BTO (spot 1)	4.00(3)	4.07(1)	1.01(1)	+0.0(1)	+0.9(2)
	BTO (spot 2)	4.00(3)	4.09(1)	1.02(1)	+0.0(1)	+1.4(2)

LSAT	LSMO	3.869(5)	3.896(9)	1.007(4)	−0.2(1)	+0.5(2)
	BTO	4.00(3)	4.10(2)	1.03(1)	+0.0(1)	+1.7(5)

STO	LSMO	3.905(7)	3.855(9)	0.987(4)	+0.7(2)	−0.5(2)
	BTO (spot 1)	4.01(2)	4.07(1)	1.015(8)	+0.3(2)	+0.9(2)
	BTO (spot 2)	4.01(2)	4.09(1)	1.020(8)	+0.3(2)	+1.4(2)

**Figure 2:**
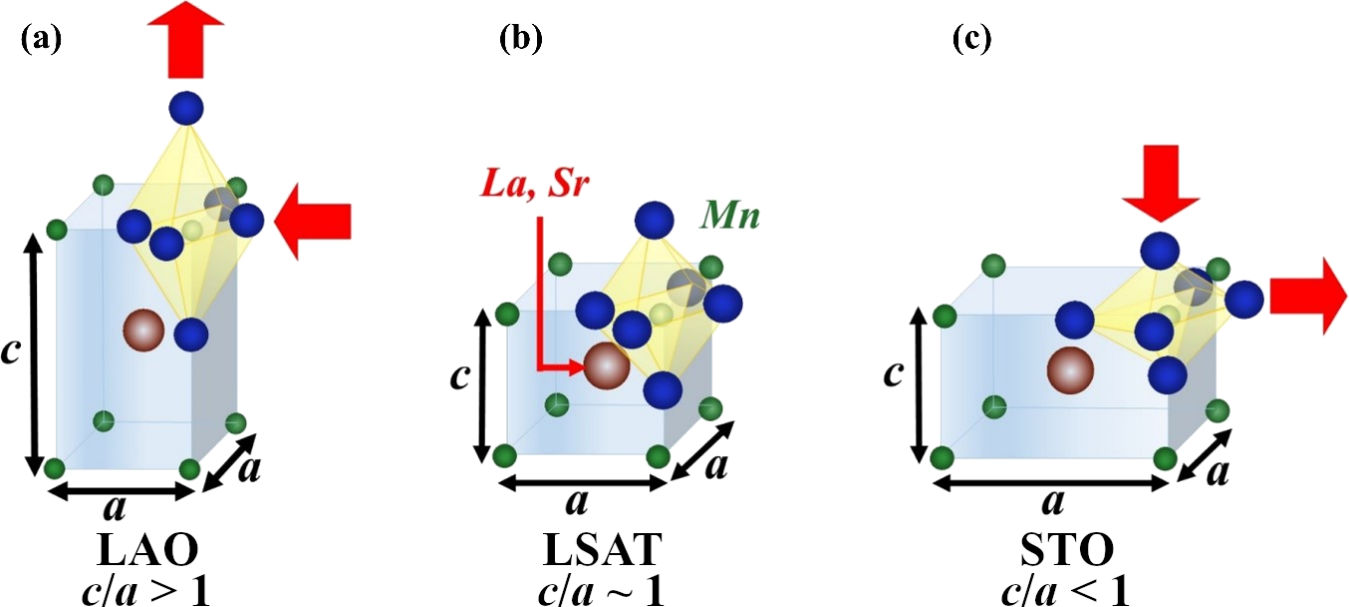
Sketch of lattice distortion of the LSMO crystal cell induced by the substrate: (a) compressive–strain deformation on LAO, (b) nearly unstrained growth on LSAT, and (c) tensile-strained deformation on STO.

Therefore, we have three scenarios that depend on the crystal distortion of the LSMO film: 1) a bilayer with a compressive-strained LSMO film on LAO substrate; 2) a bilayer with a weakly compressive-strained LSMO film on LSAT substrate and 3) a bilayer with a tensile-strained LSMO film on STO substrate. Figure 2 displays a scheme illustrating each case.

**Figure 3:**
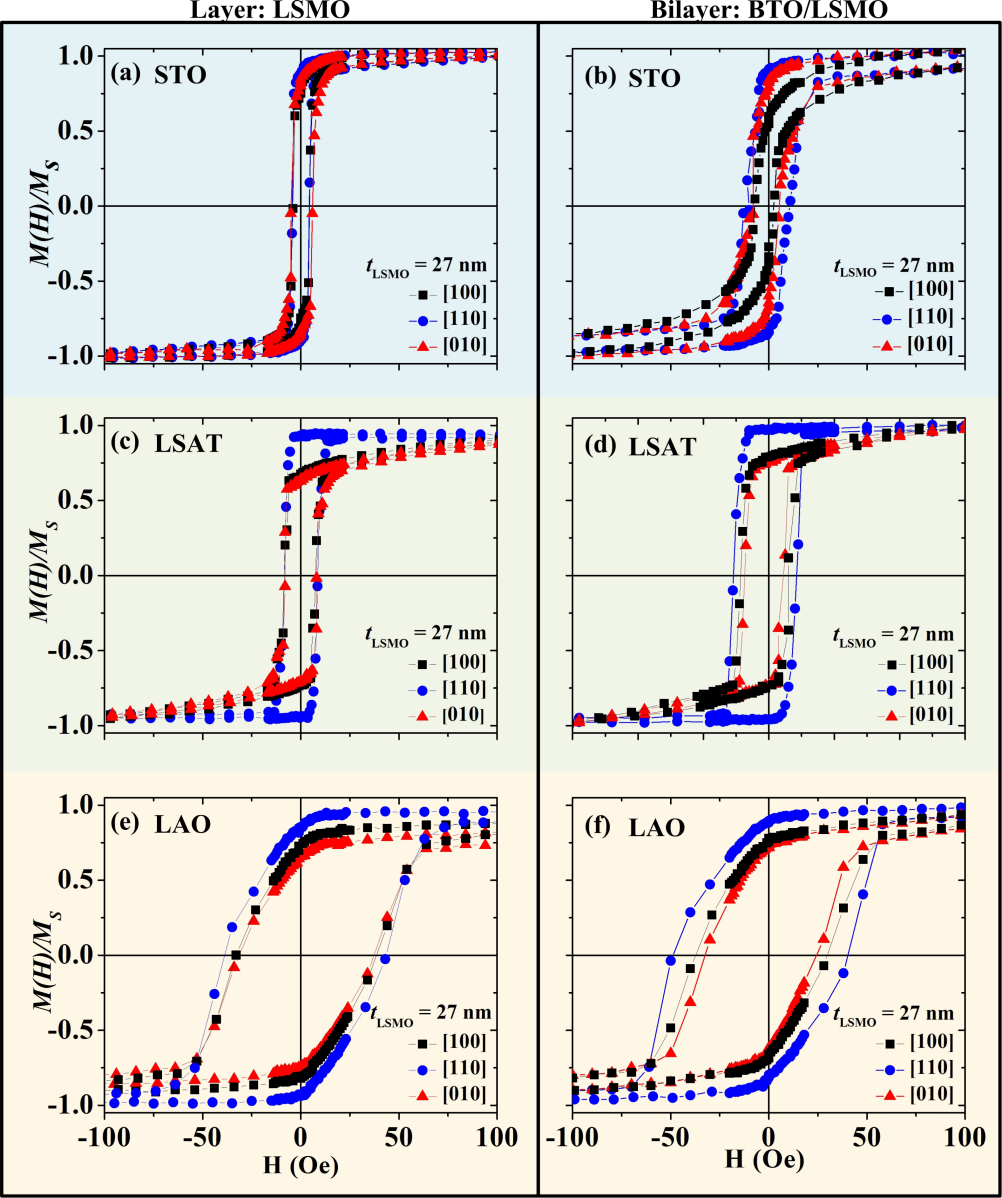
Normalized isothermal hysteresis loops at 300 K for LSMO films grown on (a) STO, (c) LSAT and (e) LAO substrates, and for BTO/LSMO bilayers grown on (b) STO, (d) LSAT and (f) LAO substrates with applied field along [100] (black squares), [010] (red triangles), and [110] (blue circle) in-plane directions.

Figure 3 displays isothermal room temperature loops of the normalized magnetization (*M*(*H*)/*M*_s_) as a function of the applied magnetic field for 27 nm thick LSMO films (plots to the left), and for BTO (140 nm)/LSMO (27 nm) bilayers (plots to the right) grown on STO (plots on the top), LSAT (central plots), and LAO (plots at the bottom) substrates. Hysteresis loops were measured by applying the magnetic field in the plane of the film along the three high-symmetry axes: [100] (black squares), [110] (blue circle), and [010] (red triangles) directions. We observe that the magnetization loop shape depends on both substrate and the applied field direction. For the LSMO film grown on STO, there is almost no difference among the three narrow hysteresis loops (Figure 3a). For the LSMO film grown on LSAT, a narrow and nearly square-shaped loop is observed along the [110] direction (blue circles) while distorted loops due to a reduced remnant magnetization are found along the [100] (black squares) and [010] (red triangles) directions (Figure 3c). For the film grown on LAO, broad loops with progressive reversal magnetization are observed in the three directions (Figure 3e). For all LSMO single layers, a maximum value of *M*_r_ is found in the hysteresis loops taken along the [110] direction (blue circles), revealing that such direction constitutes either a magnetization easy axis, for a given in-plane anisotropy, or a magnetization intermediate axis for an out-of-plane anisotropy. For BTO/LSMO samples grown on LSAT (Figure 3d) and LAO (Figure 3f) we do not detect appreciable changes in the shape of the hysteresis loops in comparison with those for LSMO film. However, in the bilayer grown on STO (Figure 3b), the *M*_r_ for the hysteresis loop taken along the [100] (black squares) is reduced when compared with the loop measured along the [010] direction (red triangles).

**Figure 4:**
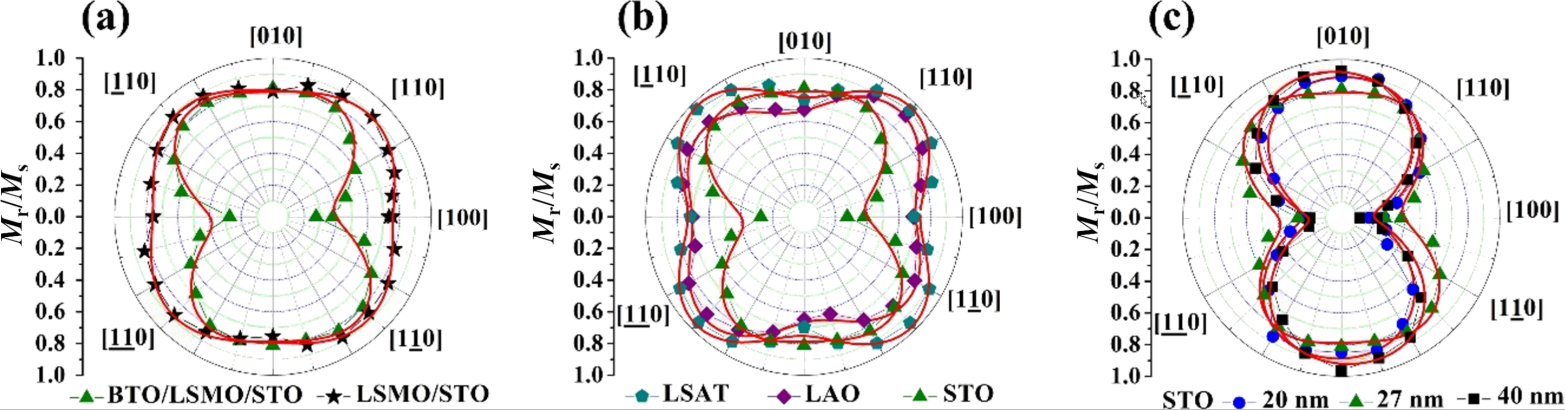
Polar plots of the normalized remnant magnetization, at 300 K, for (a) LSMO (27 nm) layer (black stars) and BTO/LSMO bilayer (green triangles) grown on STO; (b) BTO/LSMO bilayers grown on STO (green triangles), LAO (purple diamonds), and LSAT (dark cyan pentagons) substrates; (c) BTO/ LSMO bilayer with *t*_LSMO_ = 20 nm (blue circles), 27 nm (green triangles) and 40 nm (black squares). Continuous red lines correspond to numerical fits.

Figure 4 displays polar magnetization plots of *M*_r_/*M*_s_ as a function of the in-plane applied magnetic field at 300 K for a LSMO layer and for BTO/LSMO bilayers grown on different substrates and LSMO thicknesses. Figure 4a shows polar plots for a 27 nm LSMO layer (black squares) and BTO (140 nm)/LSMO (27 nm) on STO; Figure 4b BTO (140 nm)/LSMO (27 nm) bilayers grown on STO (green triangles), LAO (purple diamonds), and LSAT (blue pentagons); Figure 4c BTO (140 nm)/LSMO (*t*_LSMO_) bilayer grown on STO with *t*_LSMO_ = 20 nm (blue circles), 27 nm (green triangles) and 40 nm (black squares). For the LSMO/STO system (Figure 4a), we observe that the in-plane magnetic anisotropy shows a four-fold shape suggesting a predominant biaxial anisotropy with magnetization easy axes along four in-plane diagonal directions ([110], [110], [110] and [110]). This type of biaxial anisotropy has been observed in virtually unstrained, and tensile-strained LSMO films grown on cubic-crystal substrate [15,19,38–43]. A similar four-fold shape was also observed in single layers (not shown here) and bilayers (Figure 4b) grown on LSAT and LAO, indicating that the biaxial anisotropy is preserved after the deposition of the BTO layer.

Interestingly, the plots show how the four-fold shape in the LSMO film grown on STO (Figure 4a) is transformed into a two-fold shape in the bilayer, with in-plane easy axis along the [010] and [010] directions. Such unexpected change could reflect that a 140 nm-thick BTO layer grown onto a 27 nm-thick tensile-strained LSMO film distorts its biaxial magnetic anisotropy. Moreover, a similar measurement in bilayers (where we varied the thickness of the LSMO film from 20 to 40 nm, keeping constant the 140 nm thickness of the BTO layer, Figure 4c) proved that a predominant uniaxial anisotropy is still present in LSMO films up to 40 nm thickness.

Following a similar approach presented in [19], where the total anisotropy energy of strained LSMO films contains both biaxial and uniaxial contributions, we estimated the uniaxial (*k*_u_) and biaxial (*k*_1_) anisotropy constants for the LSMO layer and BTO/LSMO bilayer grown on STO substrate. First, we determine the anisotropy energy ratios (*k*_u_/*k*_1_) by fitting the polar plots and determining the angles that minimize the general anisotropy energy equation. To separate *k*_u_/*k*_1_, we obtain *k*_u_ by calculating the anisotropy field through the slope of the derivative of the magnetization with respect to the field (*H*) at *H* = 0 in the in-plane hard axis magnetization loop (an extended explanation is presented in [19]). For the LSMO single layers, as the four-fold shape of the polar plots suggests, an important biaxial contribution exists with an average value of 4 kJ/m^3^ and a negligible uniaxial contribution, with a value of *k*_u_ < 0.4 kJ/m^3^. For the BTO/LSMO bilayer grown on STO substrate, the presence of the BTO overlayer dramatically decreases the biaxial contribution, and both *k*_1_ and *k*_u_ reach a similar average value of 0.4 kJ/m^3^. In addition, the uniaxial contribution in the BTO/LSMO bilayer grown on STO substrate increases slightly from 0.38 kJ/m^3^ (for *t*_LSMO_ = 20 nm) to 0.55 kJ/m^3^ (for *t*_LSMO_ = 40 nm). Thus, our experimental results allow us to conclude that the deposition of BTO in the top layer of the tensile-strained LSMO drastically changes the magnetic anisotropy of the ferromagnetic layer.

In previous studies, an emergent uniaxial contribution in LSMO films grown on (001)-oriented STO is associated with crystal distortions of the film where the tetragonal crystal structure of the tensile-strained film can be locally altered with the formation of an orthorhombic structure due to different rotation patterns of the MnO_6_ octahedra to favor epitaxial growth [44–45]. In most of the cases, this is promoted by the substrate surface morphology (e.g., regular step-terrace structures, large miscut angle or lithographed periodic stripes) [15,17–18,45]. To observe if the large mismatch between the tensile-strained LSMO and BTO systems (≈2.35%) results in deformation of the LSMO atomic layers close to their interface, we performed a local strain study.

**Figure 5:**
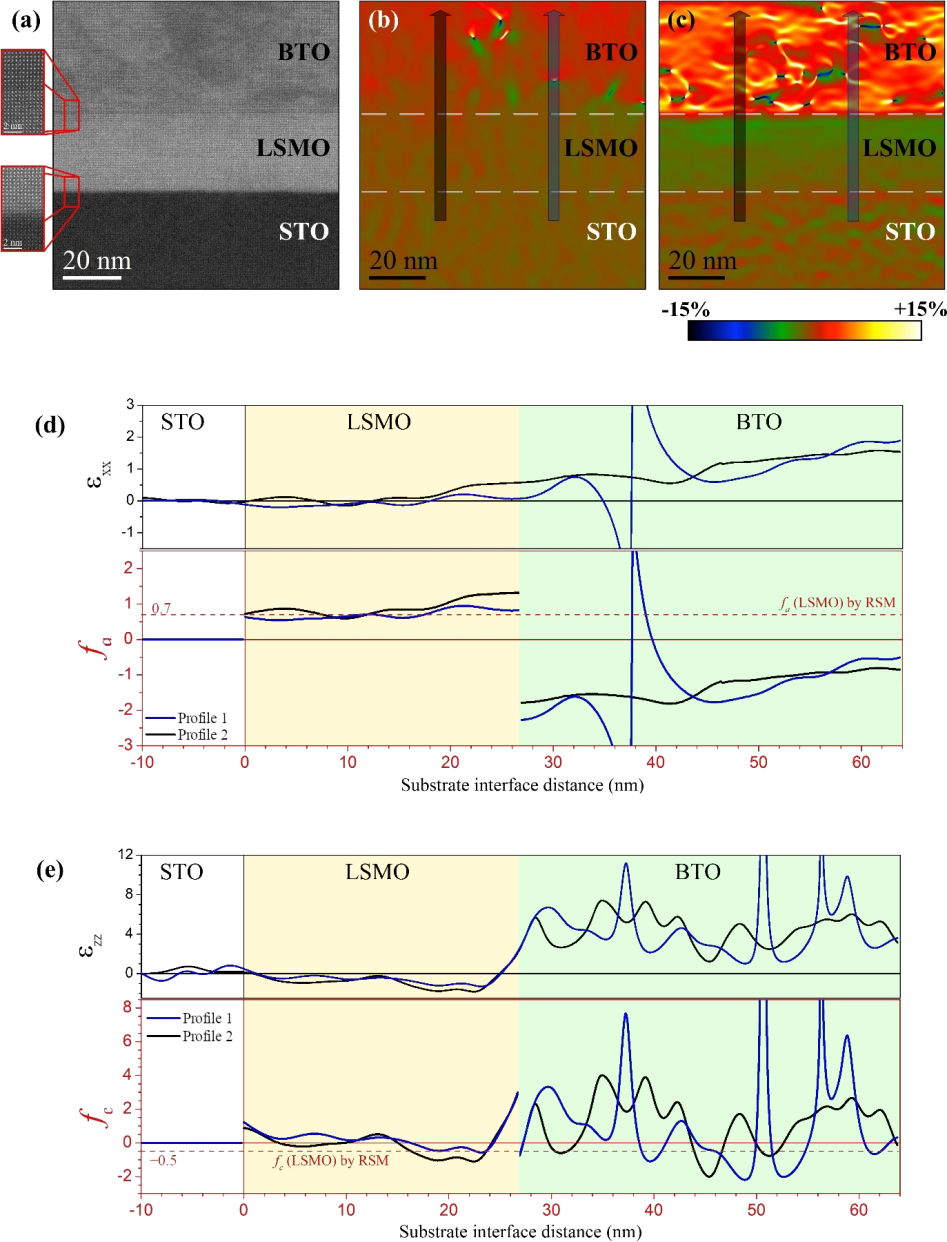
(a) Cross-section HAADF-STEM image for a BTO/LSMO bilayer grown on STO. Insets correspond to high-magnification HAADF-STEM images recorded close to the BTO/LSMO and LSMO/STO interfaces, top and bottom, respectively. Strain maps for the (b) in-plane, ε_xx_, and (c) out-of-plane, ε_zz_, lattice parameters obtained by applying GPA to (a). Dotted white lines mark the interfaces. (d) In-plane [ε_xx_ and *f*_a_] and (e) out-of-plane [ε_zz_ and *f*_c_] vertical strain profiles extracted from the GPA maps. Black and blue arrows mark the profile direction and area. Profile 1 (blue line) passes through a misfit BTO dislocation, and Profile 2 (black line) passes through a dislocation-free region.

Figure 5a displays an HAADF-STEM image for a BTO/LSMO bilayer grown on STO substrate, where we can identify both BTO/LSMO and LSMO/STO interfaces. The insets correspond to high-magnification HAADF-STEM images to highlight the flat atomic sharp interfaces. By means of GPA method on the HAADF images, it is possible to display the in-plane deformation maps (ε_xx_), Figure 5b, and out-of-plane deformation maps (ε_zz_), Figure 5c. The GPA strain maps are reconstructed considering a certain crystalline region as reference (in this case, the STO lattice) so they provide information about the relative difference (or relative strain) of the lattice parameters (in percentage) between a certain crystal phase and the lattice reference [46]. Such relative differences are calculated as ε_xx_ = [100 × (*a*_film-system_ − *a*_STO_)/*a*_STO_] and ε_zz_ = [100 × (*c*_film-system_ − *c*_STO_)/*c*_STO_], where *a*_STO_ and *c*_STO_ correspond to the in-plane and out-of-plane lattice parameters of the STO substrate. We can then relate the in-plane lattice deformation *f*_a-system_ and in-plane deformation maps ε_xx_, as well as the out-of-plane strain *f*_c-system_ and out-of-plane deformation maps ε_zz_*,* by means of the following equations:

[3] $$f_{\text{a-system}}=\frac{a_{\text{STO}}\left(\varepsilon_{\text{xx}}+100\right)}{a_{\text{bulk-system}}}-100$$

[4] $$f_{\text{c-system}}=\frac{c_{\text{STO}}\left(\varepsilon_{\text{zz}}+100\right)}{c_{\text{bulk-system}}}-100$$

The local color variations observed in the strain maps reflect homogeneous and dislocation-free STO and LSMO layers, and a BTO layer with several linear defects, most of which are concentrated close to the BTO/LSMO interfaces. Misfit dislocation-free epitaxial growth of LSMO films under (001)-oriented STO substrates is expected and widely reported [2,47–50] due to their small mismatch (≈0.7%); dislocations appear in LSMO under compressive strain [51–53], while twin walls are expected under tensile strain [54–56] with the thickness-dependent elastic deformations already in LSMO/STO systems [54]. Linear defects in the BTO layer are promoted by the large mismatch between the lattice parameters of the BTO and the strained LSMO film (≈2.4%). They correspond to misfit dislocations created parallel and perpendicular to the interface and favor the relaxation of the BTO atomic layers placed far from the BTO/LSMO interface.

For a quantitative strain analysis, profiles of ε_xx_ and ε_zz_ extracted from the GPA maps are plotted in Figure 5d and Figure 5e. These profiles are traced perpendicular to the interfaces, along two zones: Through a horizontal misfit dislocation (Profile 1) and through a dislocation-free region (Profile 2). For comparison between the strain results obtained by GPA and by RSMs, we also plotted strain profiles in terms of *f*_a-system_ and *f*_c-system_.

In Profile 1, we see that ε_xx_ is equal to zero, on average, inside the STO and LSMO systems which presents a discontinuity around the BTO dislocation and finally follows an increasing tendency, reaching a value of 1.54% at 37 nm thickness of BTO (upper limit of the maps of Figure 5a) and a maximum value of 2.27% at the BTO surface (see strain profile in the Supporting Information File 1). A zero value of ε_xx_ between STO and LSMO is caused by adapting *a*_film-LSMO_ with *a*_STO_, while the increasing behavior of ε_xx_ observed above the dislocation is associated with the tendency of BTO to recover its unstrained in-plane lattice parameter in bulk.

A different behavior is observed in Profile 2, particularly in the LSMO layer where ε_xx_ seems to not change in the first 16 nm approximately (with an average value of 0.0%) to then progressively increase until 0.57%. This fact evidences that the BTO layer locally induces an additional tensile deformation to *a*_film-LSMO_, up to twice that already induced by the substrate. This extra elongation of *a*_film-LSMO_ seems to help the relaxation of the BTO layer, hence horizontal misfit dislocations are not formed. Similar profiles were extracted from the ε_zz_ strain maps. Compared to the ε_xx_, the ε_zz_ profiles present noisier behavior inherent to the GPA method [57], with an extended and strong variation around each interface that does not allow measuring the strain close to them (around 2 nm above and below the interface). However, valuable information can be extracted from them, especially in the LSMO layer where we see that the ε_zz_ profiles follow a decreasing trend, reveling that the out-of-plane LSMO lattice parameter tends to shrink, as expected in tensile-strained films. In terms of *f*_a_ and *f*_c_, the profiles clearly show that substrate-induced tensile strain elongates the in-plane LSMO lattice parameter around 0.7% (which agrees with that calculated from the RSM of Figure 1c). A local extra elongation (*f*_a_ up to 1.0%) occurs close to the BTO/LSMO interface, in regions far from the BTO misfit dislocations, while the out-of-plane LSMO lattice parameter is slightly elongated (by around 0.3%) at the substrate interface due to the suppression of octahedral rotations in the film [35]. This elongation then shrinks between 0.5% (in agreement with that calculated from the RSM of Figure 1c) and 1.0%, in the region far from BTO misfit dislocations.

As discussed, shrinkage of *c*_film-LSMO_ is a direct consequence of the tensile-induced strain effect provoked by the substrate in the film; however, a small expansion close to the substrate interface has been observed in the LSMO film grown on STO (001) [35] and is caused by the suppression of octahedral rotations. Again, we find that the absence of misfit dislocations close to the BTO/LSMO interface induces additional shrinkage to the out-of-plane lattice parameter of the LSMO film. Therefore, the microscopic study of the crystal strain reveals that the BTO overlayer promotes an inhomogeneous strain distribution in the LSMO atomic layers close to the BTO/LSMO interface, where the absence of BTO misfit dislocations induces additional tensile-strain effect in the surrounding LSMO lattice. Such additional strain results in *c*/*a* ratios down to 0.976 that can be much higher at the boundary of the BTO/LSMO interface. As demonstrated in a previous work [45], a drastic reduction of the *c*_film-LSMO_/*a*_film-LSMO_ ratio favors rotation of the MnO_6_ octahedra out of the plane. This results in the emergence of uniaxial anisotropy that increases with the increase of the tilting angle of the MnO_6_. Thus, the uniaxial contribution of the magnetic anisotropy in the tensile-strain LSMO films comes from these extra tensile-strained regions.

## Conclusion

In summary, our study on the magnetic anisotropy of an artificial ferroelectric BTO/LSMO system demonstrates an unexpected in-plane uniaxial magnetic anisotropy in the ferromagnetic layer. In more detail, we found that a BTO overlayer modifies the biaxial anisotropy of tensile-strained LSMO films grown on (001)-oriented STO substrates towards a uniaxial anisotropy. Such change is not observed in compressive-strained LSMO films grown on (001)-oriented LSAT and LAO substrates. A microscopy analysis of the crystal deformation determined that the BTO overlayer locally causes a non-uniform strain distribution in the LSMO atomic layers close to the BTO/LSMO interfaces. However, in some regions it provokes an additional tensile strain that, in consequence, promotes the emergence of a uniaxial anisotropy. From a magnetic point of view, this finding shows a new route to alter the magnetic behavior of the LSMO layer, while from an applicative point of view, it becomes a new parameter to consider in future studies to fully understand the magnetic-electric coupling effect in this particular hybrid multiferroic system.

## Experimental

Epitaxial LSMO single layers and BTO/LSMO bilayers were grown by the PLD technique, employing a KrF excimer laser at 248 nm pulse wavelength and 20 ns pulse duration. The films were grown on 5 × 5 × 0.5 mm^3^ commercial (001)-oriented STO, LSAT, and LAO single-crystal-polished substrates, with a miscut angle lower than 0.3°. The deposition of single LSMO films was performed at a substrate temperature of 830 °C and oxygen pressure of 400 mTorr, as described elsewhere [12,58]. This was followed by a cooling cycle between 830 and 20 °C at an oxygen pressure of 700 Torr to favor optimal oxygen stoichiometry, at a cooling rate of 10 °C/min. In the case of the bilayers, the BTO film was then deposited at 830 °C and an oxygen pressure of 3 mTorr. After BTO growth, the samples were cooled down at an oxygen pressure of 700 Torr. We chose the thicknesses of the LSMO (*t*_LSMO_) and BTO (*t*_BTO_) layers to be 27 and 140 nm, respectively. Only in the case of the BTO/LSMO bilayers grown on STO, a particular batch of samples was prepared with the thickness of the LSMO layer systematically varied with *t*_LSMO_ = 20, 27, and 40 nm, maintaining the thickness of the BTO layer constant at *t*_BTO_ = 140 nm.

The thickness of each individual layer was determined by X-ray reflectivity (not shown). The crystal structure analysis of each film was performed by means of reciprocal space maps (RSM) around the pseudocubic (103) reflection that permit measuring the in-plane (*a*) and out-of-plane (*c*) lattice parameter for each layer. Both measurements were performed in a Bruker D8 ADVANCE diffractometer using a high-resolution configuration where a four-crystal Ge (220) monochromator selects the *K*_α1_ radiation from a Cu anode, providing an X-ray beam with a wavelength of λ = 1.54056 Å.

Local analysis of the crystalline structure of the bilayers was carried out by HAADF-STEM in a probe-corrected FEI Titan Low Base 60-300 microscope operated at 300 kV with a spatial resolution below 1 Å. Local strain field maps of the bilayers were obtained by applying the GPA method on HAADF-STEM images.

We studied the in-plane magnetic anisotropy through room-temperature hysteresis loops taken at different angles between the in-plane applied magnetic field and the crystallographic directions by means of a vibrating sample magnetometer (VSM). For all samples, we subtracted the linear diamagnetic contribution from the STO substrate to plot the hysteresis loops. The direction variation of the external in-plane magnetic field was changed by physically rotating the sample, using a 15° step angle. From hysteresis loops, we extracted the remnant magnetization (*M*_r_) normalized to saturation magnetization (*M*_s_) and displayed them in a polar plot. The magnetic anisotropy dependence on the substrate for both the single LSMO layer and BTO/LSMO bilayer was studied, as well as its dependence on the thickness of the LSMO layer in the bilayer film.

## Supporting Information

This file contains two figures showing magnetization hysteresis loops performed in the BTO/LSMO/STO bilayer to prove that the magnetization easy axis has an in-plane orientation and the in-plane [ε_xx_ and *f*_a_] vertical strain profile that covers the entire thickness of the BTO layer.

File 1 Additional figures.

## References

[1] Li T X, Zhang M, Yu F J, Hu Z, Li K S, Yu D B, Yan H (2012). J Phys D: Appl Phys.

[2] Boschker H, Huijben M, Vailionis A, Verbeeck J, van Aert S, Luysberg M, Bals S, van Tendeloo G, Houwman E P, Koster G (2011). J Phys D: Appl Phys.

[3] Singamaneni S R, Fan W, Prater J T, Narayan J (2014). J Appl Phys.

[4] Ordoñez J E, Gomez M E, Lopera W, Prieto P (2013). IEEE Trans Magn.

[5] Ordoñez J E, Gómez M E, Lopera W (2016). Rev Mex Fis.

[6] Eerenstein W, Mathur N D, Scott J F (2006). Nature.

[7] Eerenstein W, Wiora M, Prieto J L, Scott J F, Mathur N D (2007). Nat Mater.

[8] Tingxian L, Kuoshe L (2014). J Appl Phys.

[9] Béa H, Gajek M, Bibes M, Barthélémy A (2008). J Phys: Condens Matter.

[10] Keshri S, Rajput S S (2014). Phase Transitions.

[11] Velev J P, Jaswal S S, Tsymbal E Y (2011). Philos Trans R Soc, A.

[12] Marín L, Rodríguez L A, Magén C, Snoeck E, Arras R, Lucas I, Morellón L, Algarabel P A, De Teresa J M, Ibarra M R (2015). Nano Lett.

[13] Zheng R K, Wang Y, Liu Y K, Gao G Y, Fei L F, Jiang Y, Chan H L W, Li X M, Luo H S, Li X G (2012). Mater Chem Phys.

[14] Vrejoiu I, Ziese M, Setzer A, Esquinazi P D, Birajdar B I, Lotnyk A, Alexe M, Hesse D (2008). Appl Phys Lett.

[15] Perna P, Rodrigo C, Jiménez E, Teran F J, Mikuszeit N, Méchin L, Camarero J, Miranda R (2011). J Appl Phys.

[16] Boschker H, Mathews M, Houwman E P, Nishikawa H, Vailionis A, Koster G, Rijnders G, Blank D H A (2009). Phys Rev B.

[17] Suzuki Y, Hwang H Y, Cheong S-W, Siegrist T, van Dover R B, Asamitsu A, Tokura Y (1998). J Appl Phys.

[18] Mathews M, Postma F M, Lodder J C, Jansen R, Rijnders G, Blank D H A (2005). Appl Phys Lett.

[19] Boschker H, Mathews M, Brinks P, Houwman E, Vailionis A, Koster G, Blank D H A, Rijnders G (2011). J Magn Magn Mater.

[20] Perna P, Rodrigo C, Jiménez E, Mikuszeit N, Teran F J, Méchin L, Camarero J, Miranda R (2011). J Phys: Conf Ser.

[21] Ziese M (2005). Phys Status Solidi B.

[22] Boschker H, Kautz J, Houwman E P, Koster G, Blank D H A, Rijnders G (2010). J Appl Phys.

[23] Kwon C, Robson M C, Kim K-C, Gu J Y, Lofland S E, Bhagat S M, Trajanovic Z, Rajeswari M, Venkatesan T, Kratz A R (1997). J Magn Magn Mater.

[24] Ranno L, Llobet A, Tiron R, Favre-Nicolin E (2002). Appl Surf Sci.

[25] Wu Y, Suzuki Y, Rüdiger U, Yu J, Kent A D, Nath T K, Eom C B (1999). Appl Phys Lett.

[26] Desfeux R, Bailleul S, Da Costa A, Prellier W, Haghiri-Gosnet A M (2001). Appl Phys Lett.

[27] Sun J Z, Abraham D W, Rao R A, Eom C B (1999). Appl Phys Lett.

[28] Borges R P, Guichard W, Lunney J G, Coey J M D, Ott F (2001). J Appl Phys.

[29] Angeloni M, Balestrino G, Boggio N G, Medaglia P G, Orgiani P, Tebano A (2004). J Appl Phys.

[30] Aruta C, Ghiringhelli G, Bisogni V, Braicovich L, Brookes N B, Tebano A, Balestrino G (2009). Phys Rev B.

[31] Pesquera D, Herranz G, Barla A, Pellegrin E, Bondino F, Magnano E, Sánchez F, Fontcuberta J (2012). Nat Commun.

[32] You L, Wang B, Zou X, Lim Z S, Zhou Y, Ding H, Chen L, Wang J (2013). Phys Rev B.

[33] Heron J T, Schlom D G, Ramesh R (2014). Appl Phys Rev.

[34] Tebano A, Aruta C, Medaglia P G, Tozzi F, Balestrino G, Sidorenko A A, Allodi G, De Renzi R, Ghiringhelli G, Dallera C (2006). Phys Rev B.

[35] Vailionis A, Boschker H, Liao Z, Smit J R A, Rijnders G, Huijben M, Koster G (2014). Appl Phys Lett.

[36] Martin M C, Shirane G, Endoh Y, Hirota K, Moritomo Y, Tokura Y (1996). Phys Rev B.

[37] Sharma S, Tomar M, Kumar A, Puri N K, Gupta V (2015). AIP Adv.

[38] Ovsyannikov G A, Petrzhik A M, Borisenko I V, Klimov A A, Ignatov Y A, Demidov V V, Nikitov S A (2009). J Exp Theor Phys.

[39] Demidov V V, Ovsyannikov G A, Petrzhik A M, Borisenko I V, Shadrin A V, Gunnarsson R (2013). J Appl Phys.

[40] Chaluvadi S K, Ajejas F, Orgiani P, Rousseau O, Vinai G, Petrov A Y, Torelli P, Pautrat A, Camarero J, Perna P (2018). Appl Phys Lett.

[41] Tsui F, Smoak M C, Nath T K, Eom C B (2000). Appl Phys Lett.

[42] Steenbeck K, Hiergeist R (1999). Appl Phys Lett.

[43] Suzuki Y, Hwang H Y, Cheong S-W, van Dover R B (1997). Appl Phys Lett.

[44] Vila-Fungueiriño J M, Bui C T, Rivas-Murias B, Winkler E, Milano J, Santiso J, Rivadulla F (2016). J Phys D: Appl Phys.

[45] Rajapitamahuni A, Zhang L, Koten M A, Singh V R, Burton J D, Tsymbal E Y, Shield J E, Hong X (2016). Phys Rev Lett.

[46] Hÿtch M J, Snoeck E, Kilaas R (1998). Ultramicroscopy.

[47] Graziosi P, Gambardella A, Calbucci M, O’Shea K, MacLaren D A, Riminucci A, Bergenti I, Fugattini S, Prezioso M, Homonnay N (2016). AIP Adv.

[48] Vafaee M, Finizio S, Deniz H, Hesse D, Zabel H, Jakob G, Kläui M (2016). Appl Phys Lett.

[49] Navickas E, Chen Y, Lu Q, Wallisch W, Huber T M, Bernardi J, Stöger-Pollach M, Friedbacher G, Hutter H, Yildiz B (2017). ACS Nano.

[50] Carreira S J, Aguirre M H, Briatico J, Weschke E, Steren L B (2018). Appl Phys Lett.

[51] Sandiumenge F, Bagués N, Santiso J, Paradinas M, Pomar A, Konstantinovic Z, Ocal C, Balcells L, Casanove M-J, Martínez B (2016). Adv Mater Interfaces.

[52] Santiso J, Roqueta J, Bagués N, Frontera C, Konstantinovic Z, Lu Q, Yildiz B, Martínez B, Pomar A, Balcells L (2016). ACS Appl Mater Interfaces.

[53] Bagués N, Santiso J, Esser B D, Williams R E A, McComb D W, Konstantinovic Z, Balcells L, Sandiumenge F (2018). Adv Funct Mater.

[54] Sandiumenge F, Santiso J, Balcells L, Konstantinovic Z, Roqueta J, Pomar A, Espinós J P, Martínez B (2013). Phys Rev Lett.

[55] Santiso J, Balcells L, Konstantinovic Z, Roqueta J, Ferrer P, Pomar A, Martínez B, Sandiumenge F (2013). CrystEngComm.

[56] Balcells L, Paradinas M, Baguès N, Domingo N, Moreno R, Galceran R, Walls M, Santiso J, Konstantinovic Z, Pomar A (2015). Phys Rev B.

[57] Zhu Y, Ophus C, Ciston J, Wang H (2013). Acta Mater.

[58] Marín L, Morellón L, Algarabel P A, Rodríguez L A, Magén C, De Teresa J M, Ibarra M R (2014). Nano Lett.

