# Supplementary material for: Observation of unexpected uniaxial magnetic anisotropy in La2/3Sr1/3MnO3 films by a BaTiO3 overlayer in an artificial multiferroic bilayer
Source: Beilstein J Nanotechnol. 2020 Apr 16;11:651–61. doi: 10.3762/bjnano.11.51 (PMC7176924; doi:10.3762/bjnano.11.51)
Supplement: File 1 — Additional figures. [file Beilstein_J_Nanotechnol-11-651-s001.pdf]

BEILSTEIN JOURNAL OF NANOTECHNOLOGY

# Supporting Information

for

# Observation of unexpected uniaxial magnetic anisotropy in $La_{2/3}Sr_{1/3}MnO_3$ films by a $BaTiO_3$ overlayer in an artificial multiferroic bilayer

John E. Ordóñez, Lorena Marín, Luis A. Rodríguez, Pedro A. Algarabel, José A. Pardo, Roger Guzmán, Luis Morellón, César Magén, Etienne Snoeck, María E. Gómez and Manuel R. Ibarra

*Beilstein J. Nanotechnol.* **2020,** *11,* 651–661. doi:10.3762/bjnano.11.51

## Additional figures

License and Terms: This is a supporting information file under the terms of the Creative Commons Attribution License (http://creativecommons.org/licenses/by/4.0). Please note that the reuse, redistribution and reproduction in particular requires that the authors and source are credited.
The license is subject to the *Beilstein Journal of Nanotechnology* terms and conditions: (https://www.beilstein-journals.org/bjnano)

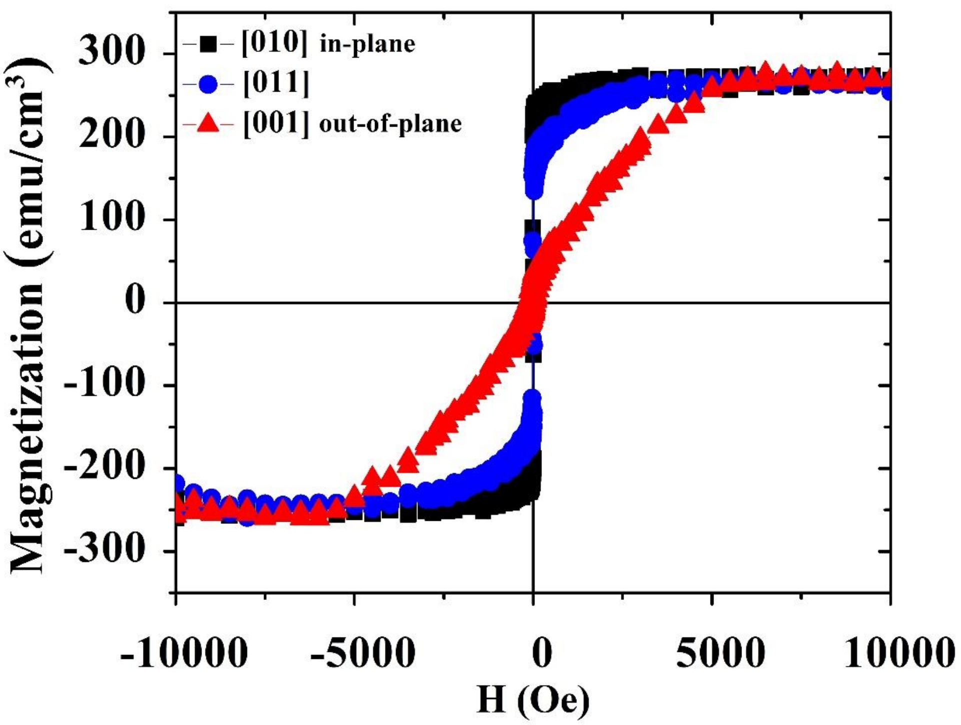


**Figure S1:** Magnetization hysteresis loops performed in the BTO/LSMO/STO bilayer to prove that the magnetization easy axis has an in-plane orientation.

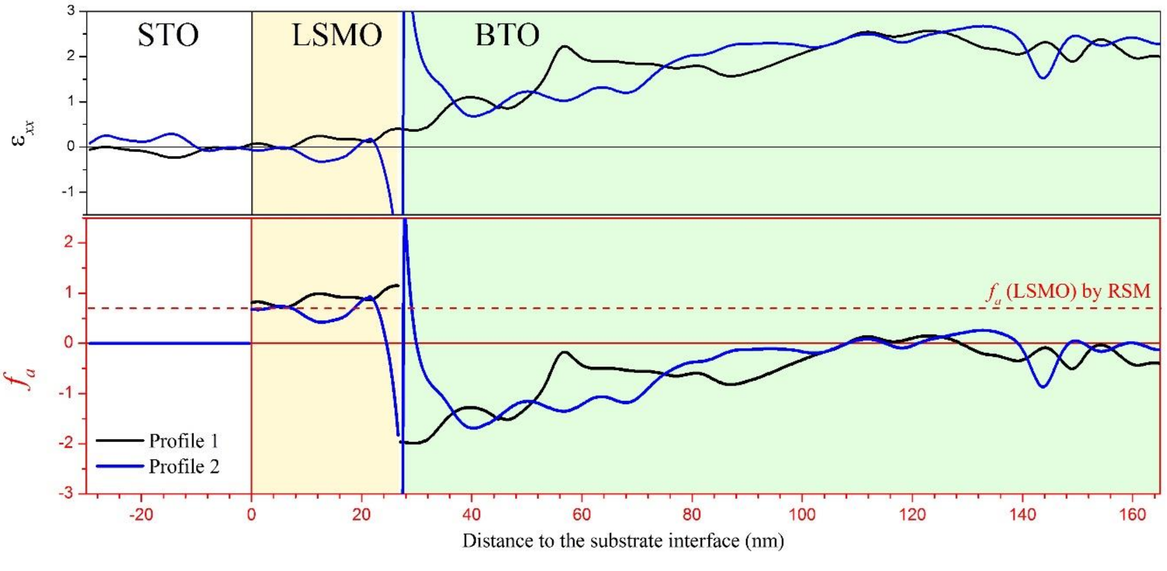


**Figure S2:** In-plane [$\varepsilon_{xx}$ and $f_a$] vertical strain profiles that cover the entire thickness of the BTO layer.
